# Supplementary figures and images for: Modeling osteoporosis to design and optimize pharmacological therapies comprising multiple drug types
Source: eLife. 2022 Aug 9;11:e76228. doi: 10.7554/eLife.76228 (PMC9363122; doi:10.7554/eLife.76228)

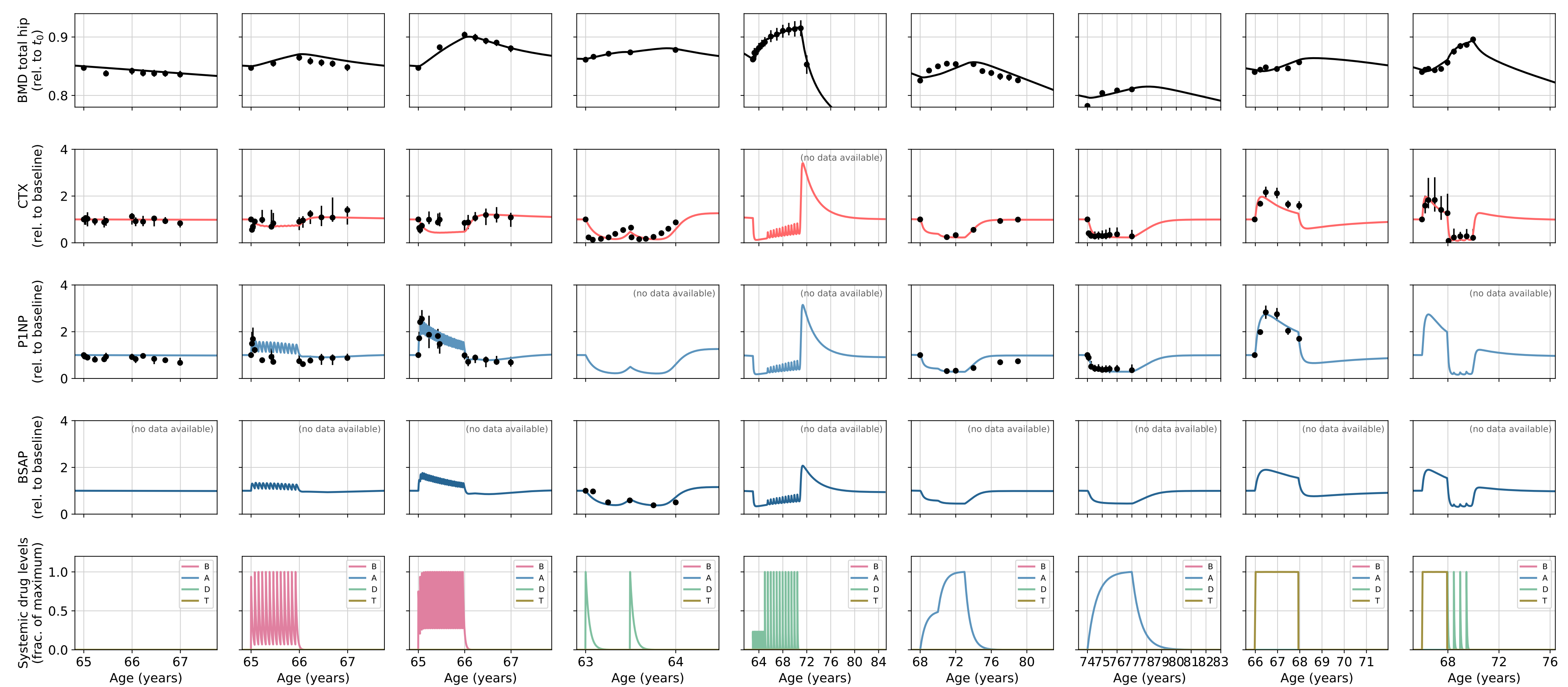

Supplement: Source code 1. [file elife-76228-code1.zip › run_scripts/results/calibration.pdf]

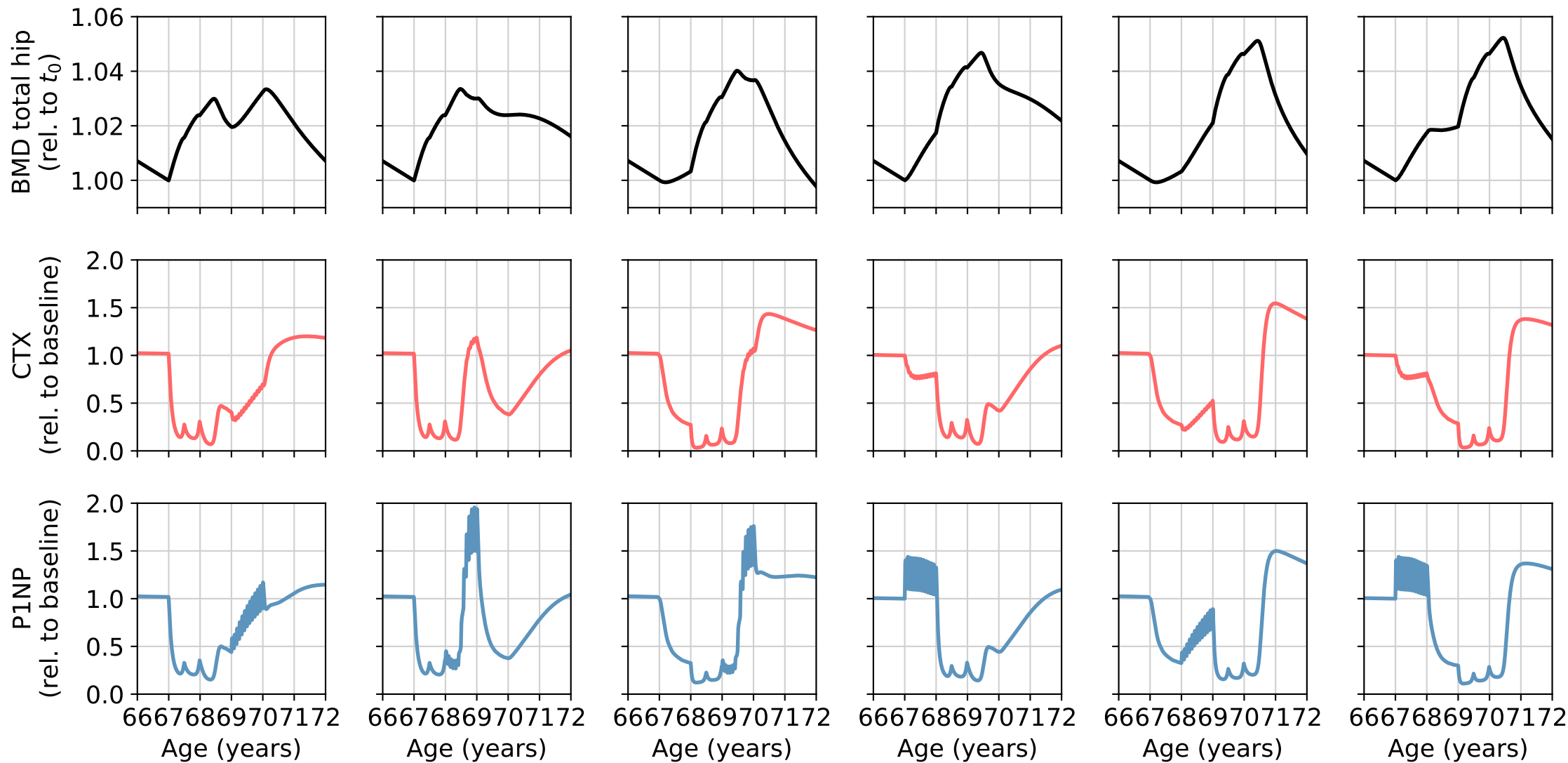

Supplement: Source code 1. [file elife-76228-code1.zip › run_scripts/results/treatment_scenario.pdf]

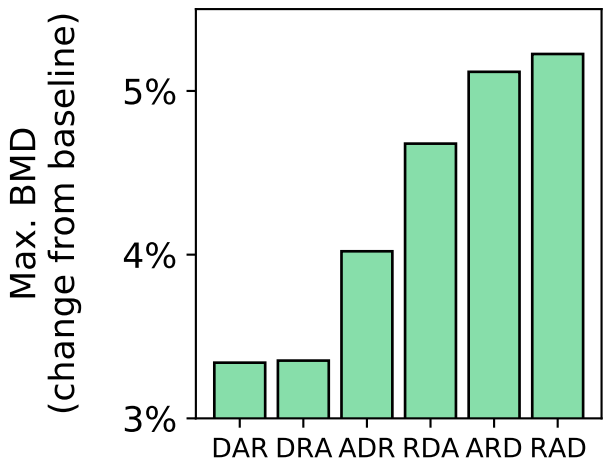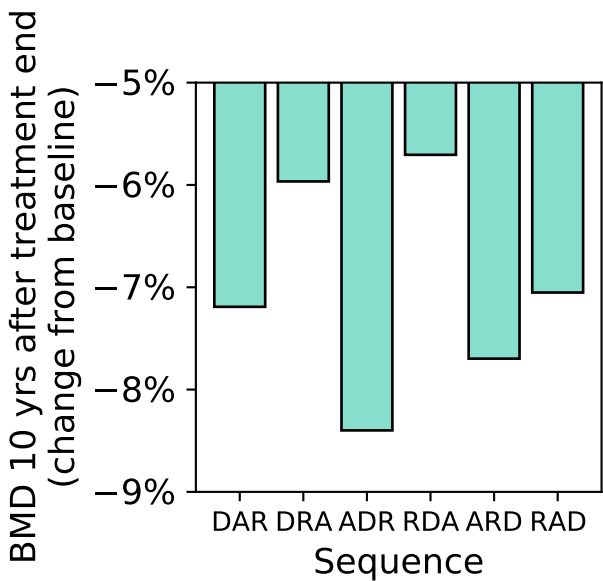

Supplement: Source code 1. [file elife-76228-code1.zip › run_scripts/results/treatment_scenario_globals.pdf]

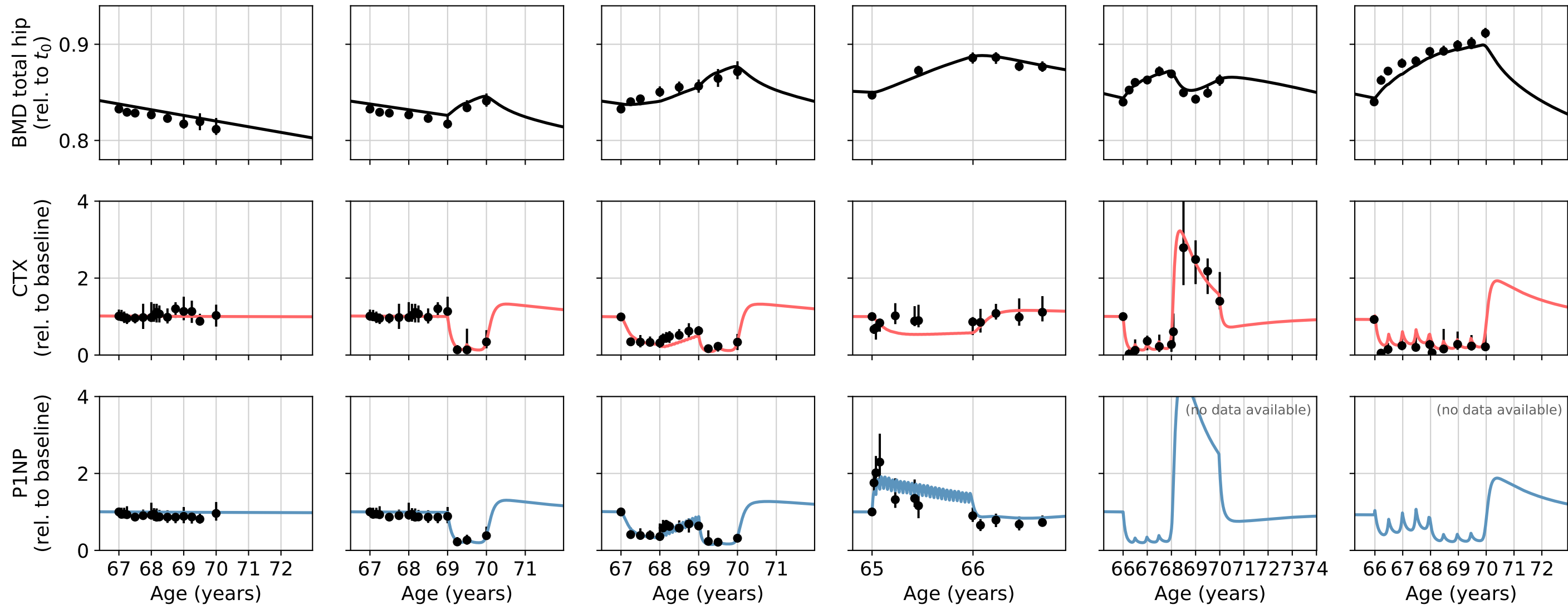

Supplement: Source code 1. [file elife-76228-code1.zip › run_scripts/results/validation_main.pdf]

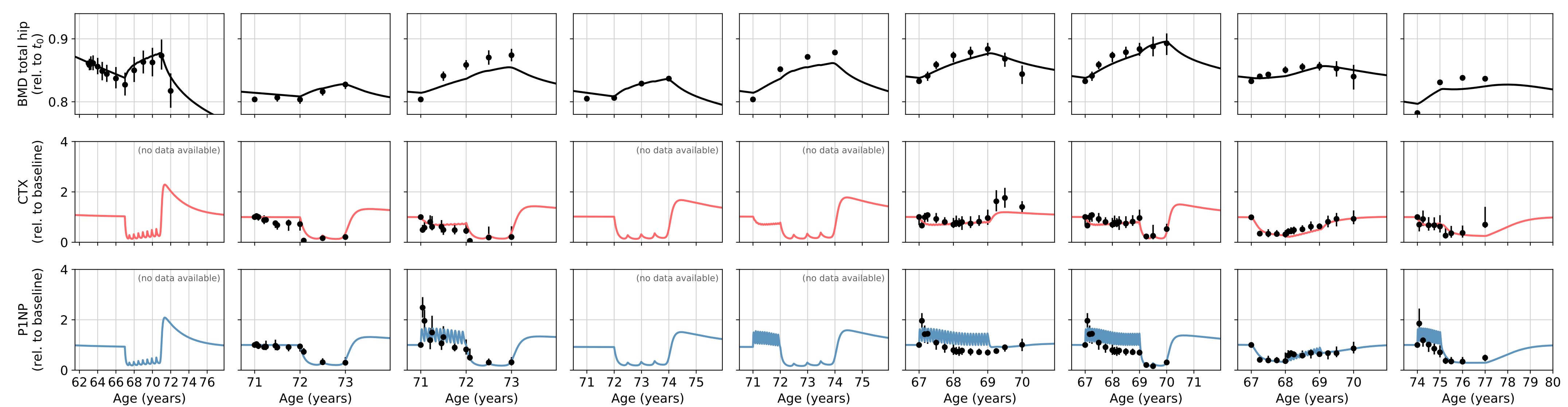

Supplement: Source code 1. [file elife-76228-code1.zip › run_scripts/results/validation_supplementary.pdf]
